# Influence of the definition of “metabolically healthy obesity” on the progression of coronary artery calcification

**DOI:** 10.1371/journal.pone.0178741

**Published:** 2017-06-02

**Authors:** Ji Won Yoon, Chan-Hyeon Jung, Min-Kyung Kim, Hyo Eun Park, Kyong Soo Park, Hak Chul Jang, Min Kyong Moon, Su-Yeon Choi, Bo Kyung Koo

**Affiliations:** 1 Department of Internal Medicine, Healthcare System Gangnam Center, Seoul National University Hospital, Seoul, Republic of Korea; 2 Department of Internal Medicine, Seoul National University College of Medicine, Seoul, Republic of Korea; 3 Department of Internal Medicine, Seoul National University Bundang Hospital, Sungnam, Republic of Korea; 4 Department of Internal Medicine, Seoul National University Boramae Medical Center, Seoul, Republic of Korea; Universita degli Studi Magna Graecia di Catanzaro Scuola di Medicina e Chirurgia, ITALY

## Abstract

**Objectives:**

Debates whether metabolically healthy obesity (MHO) increases the cardiovascular risk might be due to the metabolic instability of MHO or the absence of a perfect definition of MHO. Therefore, we aimed to investigate the influence of the MHO phenotype on the coronary artery calcium score (CACS) progression according to definition of MHO.

**Methods:**

We analyzed a retrospective cohort with a CACS of 0 at baseline and available serial CACS measurements taken ≥ 12 months apart (n = 1,218). Obesity was defined as BMI ≥ 25 kg/m^2^, and MHO was defined as obesity accompanied by ≤ 1 (MHO class I) or 0 (MHO class II) components of metabolic syndrome (MetS).

**Results:**

During a median follow-up of 45 months, 32.2% of MHO class I and 10.2% of MHO class II subjects developed MetS. Compared to non-obese/metabolically healthy subjects (reference group), hazard ratios (HR) for development of MetS were 2.174 (95% confidence interval [CI]: 1.513–3.124) and 1.166 (95% CI: 0.434–3.129) for MHO class I and II subjects, respectively. The MHO class I subjects showed a significantly increased risk of CACS progression as compared to the reference group (HR: 1.653; 95% CI: 1.144–2.390), whereas MHO class II subjects did not (HR: 1.195; 95% CI: 0.514–2.778). Among subjects with MHO class I, no significant CACS progression was observed in the subjects who maintained metabolic health during follow-up (HR: 1.448; 95% CI: 0.921–2.278).

**Conclusions:**

The risks of metabolic deterioration and CACS progression were significant in subjects with MHO class I, but not in those with MHO class II.

## Introduction

The prevalence of obesity has increased rapidly in recent years [1] and is associated with various metabolic abnormalities that lead to cardiovascular morbidity and mortality [1]. However, obesity is not always associated with metabolic abnormality. Obesity without metabolic disturbance has been reported in 20–40% of obese people [2,3]: This phenotype of obesity is defined as metabolically healthy obesity (MHO) and is not associated with the risk of atherosclerosis [4]. Previous studies have reported several possible mechanisms underlying MHO, such as difference in fat distribution [4], subclinical inflammation [5], expansion capacity of adipose tissue [6], adiponectin level [7,8] and adipose carbohydrate responsive element binding protein β [9]. However, longitudinal studies have shown that MHO is an unstable state and it progresses to a metabolically unhealthy and obese (MUHO) status in a considerable proportion of patients [10,11]. Furthermore, although individuals categorized as MHO have demonstrated increased cardiovascular events compared to non-obese individuals during long-term follow-up [12], the data are conflicting among studies [13,14].

Considering that the Asian population has a higher metabolic risk increase for the same increase in body mass index (BMI) compared to Caucasians [15], the influence of obesity on the risk of coronary artery disease (CAD) in the Asian population should be elucidated on the basis of the metabolic status. However, only a few studies have been conducted on MHO and CAD risk in the Asian population thus far.

Among methods for assessment of cardiovascular risk, the coronary artery calcium score (CACS), which is determined by computed tomography (CT), is an excellent tool for clinical measurement of the burden of CAD risk. Quantitative pathologic analysis has shown that segments with greater calcification tend to have a larger burden of atherosclerotic plaque and percent lumen stenosis [16]. Thus, CACS reflects the presence and extent of coronary atherosclerosis [17]. Furthermore, after serial assessment, CACS has been proposed as a useful predictor of cardiac outcome [18–21]. Therefore, in this study, we aimed to investigate the influence of the MHO phenotype on the development of subclinical coronary atherosclerosis by evaluating CACS in a retrospective cohort.

## Materials and methods

### Study population

This was a retrospective cohort study. Among people who underwent routine general health examinations at the Healthcare System Gangnam Center, Seoul National University Hospital (n > 10,000/year), we shortlisted 17,390 subjects who underwent coronary artery CT evaluation for screening purpose on their demand from October 2003 and December 2013.Of these, 2,473 subjects underwent baseline and follow-up coronary artery CT for assessment of CACS. Of those, we selected 2,435 subjects whose second CACS evaluation was at least 12 months after the initial evaluation. Thereafter, we excluded subjects who had (1) experienced coronary revascularization including coronary artery bypass surgery (n = 15) or (2) insufficient clinical data to define obesity and metabolic syndrome (n = 62). Further, we excluded subjects with baseline CACS > 0. Finally, 1,218 subjects (882 men and 336 women) with baseline CACS of 0 were included in the analysis.

The study protocol conformed to the ethical guidelines of the 2013 Declaration of Helsinki, and was reviewed and approved by the institutional review board (IRB) of Seoul National University Hospital (IRB No. H-1401-099-549) and the IRB of Boramae Medical Center (IRB No. 26-2013-105). As the current study was retrospective in nature and involved the use of a database and medical records, informed consent was waived by the board. All participants were informed of the possible risk of radiation exposure by CT scanning and agreed to undergo the examination.

### Clinical and laboratory assessments

Clinical and laboratory assessments were performed at baseline and follow-up. Height and body weight were measured to the nearest 0.1. BMI was calculated as body weight (kg) divided by height squared (m^2^). Waist circumference (WC) was measured mid-way between the iliac crest and the lower rib margin by a trained nurse. Systolic and diastolic blood pressures were also measured. Blood samples were taken > 12 h after fasting, and the levels of fasting blood glucose, high-density lipoprotein (HDL) cholesterol, and triglycerides (TG) were measured using Architect Ci8200 (Abbott Laboratories, Abbott Park, IL, USA). Hemoglobin A1c (HbA1c) concentration was measured using COBAS INTEGRA 400 (Roche Diagnostics GmbH, Mannheim, Germany). The low-density lipoprotein (LDL) cholesterol level was calculated by using the Friedewald equation in subjects with a TG level < 4.5 mmol/L. In subjects with a TG level ≥ 4.5 mmol/L, the measured LDL cholesterol level was used for analysis. Fasting insulin level was measured with an immunoradiometric assay kit (INS-IRMA kit; Biosource, Belgium) in part of the subjects (n = 640); and insulin resistance was indirectly evaluated using the homeostasis model assessment of insulin resistance (HOMA-IR), as described previously [22]. Subjects were asked to complete a structured questionnaire that included medical history, current medications, and smoking history at the time of the medical examinations. Hypertension was defined as blood pressure ≥140/90 mmHg or intake of antihypertensive medications. Diabetes was defined as a fasting glucose level ≥7 mmol/L or HbA1c concentration ≥ 6.5%, or intake of anti-diabetic medications. Hypercholesterolemia was defined as an LDL cholesterol level ≥ 4.1 mmol/L or intake of lipid-lowering agents.

Overweight and obesity were defined as BMI 23–24.9 kg/m^2^ and ≥ 25 kg/m^2^, respectively according to the World Health Organization Asia–Pacific criteria [23]. Abdominal obesity was defined as WC ≥ 90 cm for men and ≥ 80 cm for women according to the International Obesity Task Force criteria for the Asian-Pacific population [23]. MHO was defined according to the most frequently used definition in recent studies (MHO class I) [24–27]. Briefly, obese participants who met ≤ 1 of the following National Cholesterol Education Program–Adult Treatment Panel III (NCEP–ATP III) criteria for metabolic syndrome [28] were considered to have MHO class I: (a) TG level ≥ 1.7 mmol/L (150 mg/dL); (b) HDL cholesterol level < 1.0 mmol/L (40 mg/dL) in men and < 1.3 mmol/L (50 mg/dL) in women; (c) blood pressure ≥ 130/85 mmHg or intake of antihypertensive medication; and (d) fasting glucose level ≥ 5.6 mmol/L (100 mg/dL) or intake of anti-diabetic medication. The WC criterion was not used in the definition of metabolic health as in the previous studies [24–27,29,30], because of collinearity with BMI. Additionally, we defined MHO using stricter criteria following the Healthy Obese Project, which was a population-based cohort study conducted in 7 European countries (MHO class II) [31]: MHO class II was established when subjects with obesity had none of the metabolic syndrome components. Also, the WC criterion was not used in the definition of MHO class II. Smoking status was classified as current smokers and non-smokers. Subjects were classified as physically active if they performed regular exercise more than once per week.

### Cardiac computed tomography and analysis for CACS

Coronary CT was performed using either a 256-slice multidetector CT scanner (Brilliance iCT 256; Philips Medical Systems, Cleveland, Ohio) or a 16-slice scanner (Somatom Sensation 16; Siemens Medical Solutions, Forchheim, Germany). A standard scanning protocol was applied, with 128 × 0.625 mm section collimation, 0.27 ms rotation time, 120 kV tube voltage, and 800 mA tube current. All scans were performed with electrocardiogram-gated dose modulation. The CACS was calculated quantitatively according to the method described by Agatston et al. [32] and using a software program (Rapidia 2.8; INFINITT, Seoul, Republic of Korea). Progression of CACS was defined as any increase in CACS at follow-up according to the previous study [33]. We included only the subjects with CACS = 0 at baseline; progression was defined as CACS >0 at follow-up.

### Statistical analysis

To compare the clinical characteristics according to obesity and metabolic health status, ANOVA was used for continuous variables and χ^2^ tests for categorical variables. The Bonferroni post-hoc analysis was performed for ANOVA. Cox proportional hazard regression was used to assess the risk of developing metabolic syndrome or CACS progression during the follow-up according to the baseline obesity and metabolic status. *P*-values <0.05 were considered statistically significant. All statistical analyses were conducted using SPSS 19 (SPSS Inc., Chicago, IL, USA).

## Results

### Clinical characteristics according to metabolic abnormalities

We analyzed the cohort data from 1,218 subjects (median follow-up duration, 45 months; interquartile range, 28–59 months). Prevalence of MHO class I and class II was 15.6% (17.8% in men and 9.8% in women) and 4.8% (5.0% in men and 4.2% in women) in the study subjects, respectively. Among obese subjects, 38.9% had MHO class I and 11.9% had MHO class II.

The clinical characteristics of subjects according to the baseline obesity status and metabolic abnormalities were shown in Table 1. Irrespective of the definition of MHO, subjects with MHO group showed a significantly higher BMI, WC, blood pressure, and serum triglyceride level than metabolically healthy non-obese (MHNO) group (Table 1). However, in the case of HOMA-IR and hsCRP level, only the subjects defined as MHO class I had significantly higher levels compared to MHNO (*P* < 0.001 in both). There was no difference in HOMA-IR or hsCRP level between MHO class II and MHNO (Table 1).

**Table 1:** Clinical characteristics according to baseline metabolic abnormality and obesity.

		MHO class I						MHO class II					
		MHNO	MHO	MUHNO	MUHO	*P*-value^d^	*P*-value^e^	MHNO	MHO	MUHNO	MUHO	*P*-value^d^	*P*-value^e^
N		492	190	238	298			218	58	512	430		
Male, %		62.4	82.6	70.6	83.9	<0.001	<0.001	59.2	75.9	67.6	84.4	<0.001	0.022
Age, years		54.6± 6.4	54.6±6.8	55.9 ± 6.7	54.2 ± 7.0	0.023	0.907	54.3± 6.6	54.6 ± 6.8	55.4 ± 6.5	54.3 ± 6.9	0.055	0.758
BMI*, kg/m^2^		22.4± 1.7	26.5± 1.3	23.1 ± 1.5	27.1 ± 1.9	<0.001	<0.001	22.3±1.8	26.4± 1.2	22.8 ± 1.6	27.0 ± 1.7	<0.001	<0.001
WC*, cm	M	84.2 ± 4.7	92.3± 4.4	85.5 ± 4.7	93.6 ± 5.6	<0.001	<0.001	84.3±5.1	92.4±4.7	84.8 ± 4.6	93.2 ± 5.2	<0.001	<0.001
	F	80.1± 6.0	91.1± 5.8	83.6 ± 5.5	92.7 ± 5.4	<0.001	<0.001	79.1±5.8	89.1±4.1	82.1 ± 6.0	92.6 ± 5.7	<0.001	<0.001
Systolic BP, mmHg		113.7±13.4	119.3±13.0	125.2±13.7	127.6±13.2	<0.001	<0.001	109.1±10.2	112.6± 8.7	121.0± 14.7	125.9± 13.5	<0.001	0.019
Diastolic BP, mmHg		74.7± 10.5	78.1± 9.6	82.1 ± 10.0	85.0 ± 10.9	<0.001	<0.001	70.5± 8.3	72.9± 6.8	79.9 ±10.6	83.6 ± 10.7	<0.001	0.020
FPG, mmol/L		5.3± 0.9	5.3± 0.6	6.2 ± 1.1	6.2 ± 1.1	<0.001	0.561	5.0±0.3	5.1± 0.3	5.8 ± 1.2	6.0 ± 1.1	<0.001	0.011
HbA1c, %		5.7± 0.6	5.7± 0.4	6.0 ± 0.7	6.0 ± 0.8	<0.001	0.335	5.6 ± 0.3	5.6± 0.3	5.9 ± 0.7	5.9 ± 0.7	<0.001	0.366
LDL-C, mmol/L		3.23± 0.77	3.25 ± 0.83	3.15 ± 0.80	3.26 ± 0.85	0.397	0.763	3.16± 0.75	3.48± 0.82	3.22 ± 0.79	3.23 ± 0.84	0.064	0.005
HDL-C, mmol/L	M	1.37 ± 0.28	1.35 ± 0.29	1.27 ± 0.32	1.19 ± 0.28	<0.001	0.283	1.39 ± 0.26	1.39± 0.27	1.32± 0.31	1.24± 0.29	<0.001	0.800
	F	1.61 ± 0.34	1.44± 0.26	1.36 ± 0.38	1.28 ± 0.26	<0.001	0.002	1.66±0.33	1.48± 0.17	1.47 ±0.38	1.32 ±0.28	<0.001	0.003
Triglyceride, mmol/L*		1.04± 0.50	1.21± 0.59	1.73 ± 1.13	1.92 ± 1.00	<0.001	<0.001	0.92±0.33	1.04±0.33	1.41 ±0.93	1.73 ± 0.96	<0.001	0.012
AST*, IU/L		24.1± 10.0	26.3 ± 10.8	25.0 ± 10.6	27.9 ± 12.2	<0.001	0.002	24.4 ± 12.7	26.3 ± 12.5	24.9 ± 9.0	27.4 ±11.6	<0.001	0.105
ALT*, IU/L		23.7 ± 12.3	31.1 ± 19.3	28.0 ± 16.6	34.8 ± 22.1	<0.001	<0.001	22.3 ±9.8	30.2 ± 21.4	26.3 ± 15.2	33.8 ± 21.1	<0.001	<0.001
GGT*, IU/L		29.2 ± 30.0	40.2 ± 30.7	41.1 ± 37.7	54.1 ± 55.4	<0.001	<0.001	26.3 ± 24.1	31.5 ± 16.5	35.9 ± 34.4	51.0 ± 50.1	<0.001	0.005
HOMA-IR*		1.85± 0.87	2.41± 1.39	2.77 ± 1.29	3.36 ± 1.61	<0.001	<0.001	1.70±1.76	2.06± 1.39	2.37 ±1.19	3.13 ±1.58	<0.001	0.162
hs-CRP*, mg/L		1.1 ± 3.4	1.8± 4.0	1.1 ± 1.7	2.0 ± 4.3	<0.001	<0.001	1.1 ± 2.5	1.4 ± 2.4	1.1 ± 3.1	2.0 ± 4.3	<0.001	0.249
Diabetes mellitus, %^a^		6.5	3.2	24.4	22.1	<0.001	0.096	0.0	0.0	17.6	16.7	<0.001	NA
Hypertension, %^b^		16.5	22.6	53.4	58.1	<0.001	0.076	0.0	0.0	40.6	50.2	<0.001	NA
Abdominal obesity, %^c^		26.8	76.8	36.3	79.9	<0.001	<0.001	27.1	78.2	31.1	78.7	<0.001	<0.001
Current smoker, %		14.0	17.8	17.8	21.6	0.028	0.311	15.2	28.2	15.4	19.0	0.553	0.098
Exercise, %		29.1	30.0	34.9	33.2	0.121	0.851	25.7	22.4	33.2	33.3	0.026	0.733

*Log-transformed when comparing among groups

^a^Subjects with FPG ≥ 7.0 mmol/L, HbA1c ≥ 6.5%, or taking anti-diabetic medication

^b^Subjects with SBP ≥ 140 mmHg or DBP ≥ 90 mmHg, or taking anti-hypertensive medication

^c^Subjects with waist circumference ≥ 90 cm in men and ≥ 80 cm in women.

^d^*P* from ANOVA among 4 groups (MHNO, MHO, MUHNO, and MUHO)

^e^Compared between MHNO and MHO

Abbreviations: MHO, metabolically healthy obesity; MHNO, metabolically healthy non-obesity; MUHNO, metabolically unhealthy non-obesity; MUHO, metabolically unhealthy obesity; BMI, body mass index; WC, Waist circumference; M, male; F, female; BP, blood pressure; FPG, fasting plasma glucose; LDL-C, low density lipoprotein cholesterol; HDL-C, high density lipoprotein cholesterol; AST, aspartate aminotransferase; ALT, alanine aminotransferase; GGT, gamma-glutamyl transferase; HOMA-IR, homeostatic model assessment of insulin resistance; CRP, c-reactive protein.

To elucidate the difference between MHO class I and II, we compared obesity with no metabolic abnormalities and obesity with one metabolic abnormality. Compared to MHO subjects without any metabolic abnormality (that is, MHO class II), MHO subjects with one metabolic abnormality showed a higher HOMA-IR level (*P* = 0.015; S1 Table).

### Risk for the development of metabolic syndrome during follow-up

The further analysis for the risk of developing metabolic syndrome during the follow-up period, only the subjects with metabolic health at the baseline were included. Among obese subjects without any metabolic abnormality at baseline (MHO class II), 10.2% developed metabolic syndrome during the follow-up period, which was not significantly different as compared to 10.8% of non-obese subjects with no metabolic abnormality (age- and sex-adjusted hazard ratio [HR]: 1.166, 95% confidence interval [CI]: 0.434–3.129, *P* = 0.761; Table 2). In contrast, following the MHO class I definition, 31.6% of MHO and 16.7% of MHNO developed metabolic syndrome during the follow-up. The risk of developing metabolic syndrome in MHO class I was significantly higher than that in MHNO (age- and sex-adjusted HR: 2.174, 95% CI: 1.513–3.124, *P* < 0.001; Table 2). The prevalence of metabolic syndrome at follow-up increased linearly with the number of metabolic abnormalities at baseline (*P* for trend <0.001; S1 Fig).

**Table 2:** Risk of developing metabolic syndrome during follow-up according to baseline metabolic abnormalities and obesity status.

	Metabolic syndrome^a^ at follow-up			
	Hazzard ratio^b^	*P*-value^b^	Hazzard ratio^c^	*P*-value^c^
MHO definition I				
MHNO (≤1 abnormality)	(reference)	–	(reference)	–
Obesity with ≤1 abnormality (MHO class I)	2.359(1.650–3.374)	<0.001	2.174(1.513–3.124)	<0.001
MHO definition II				
MHNO (no abnormality)	(reference)	–	(reference)	–
Obesity with no abnormality (MHO class II)	1.197 (0.449–3.195)	0.719	1.166 (0.434–3.129)	0.761
Obesity with one abnormality	4.182 (2.482–7.045)	<0.001	4.030 (2.338–6.947)	<0.001
Obesity with ≤1 abnormality (MHO class I)	3.401 (2.033–5.690)	<0.001	3.238 (1.901–5.515)	<0.001

^a^those who met ≥ 2 of the following National Cholesterol Education Program–Adult Treatment Panel III criteria except abdominal obesity criterion

^b^ without adjustment

^c^ with adjustment for age and sex

Abbreviations: MHO, metabolically healthy obesity; MHNO, metabolically healthy non-obesity

To differentiate the risk from MHO class I and II, we subsequently analyzed the risk of developing metabolic syndrome in MHO subjects according to the number of baseline metabolic abnormality, that is, 0 or 1. We showed that there was no difference in the risk of developing metabolic syndrome between obese subjects with no metabolic abnormality (MHO class II; Table 2) and non-obese subjects with no metabolic abnormality. By contrast, obese subjects with one metabolic abnormality showed about 4 time higher risk of developing metabolic syndrome compared to MHNO subjects even after adjustment for age and sex (HR: 4.030, 95% CI: 2.338–6.947, *P* < 0.001; Table 2). Additional adjustment for HOMA-IR did not attenuated the statistical significance in that (HR: 4.186, 95% CI: 1.699–10.314, *P* = 0.002).

Subsequently, as current definition of MHNO includes overweight, which might underestimate the risk of MHO class II, we redefined the reference group as subjects with both of normal weight and metabolic health. Redefinition of reference group also confirmed a difference between MHO class I and II: only MHO class I showed a significant risk of future metabolic syndrome compared to reference group (S2 Table). MHO class II still showed no difference in the risk of developing metabolic syndrome compared to metabolically healthy and normal weight group (age and sex-adjusted HR: 1.509, 95% CI: 0.487–4.681; S2 Table).

Among subjects with MHO class I at baseline, those who showed deteriorated metabolic health at the follow-up had a significantly higher systolic and diastolic blood pressures, triglyceride, and gamma-glutamyl transferase (GGT) levels (*P* = 0.002, *P* <0.001, *P* = 0.040, and *P* = 0.006, respectively; S3 Table). Prevalence of abdominal obesity, serum CRP level and HOMA-IR were not significantly different between two groups in MHO class I (S3 Table).

### Risk for the progression of CACS

The prevalence of CACS progression during the follow-up period was 11.5%, 19.3%, 28.6%, 32.7%, and 33.3% in non-obese subjects with 0, 1, 2, 3, and 4 metabolic abnormalities, respectively (*P* for trend <0.001), and 12.1%, 29.5%, 25.3%, 37.9%, and 28.0% in obese subjects with 0, 1, 2, 3, and 4 metabolic abnormalities, respectively (*P* for trend = 0.018; S2 Fig). For the analysis assessing the risk of CACS progression according to MHO status, we only used the subjects with metabolic health at the baseline.

After adjustment for age and sex, MHO class I increased the risk of CACS progression during the follow-up period (age- and sex-adjusted HR: 1.653, 95% CI: 1.144–2.390, *P* = 0.007; Table 3). However, MHO class II did not increase the future risk of CACS progression as compared to the non-obese subjects without any metabolic abnormality (age and sex-adjusted HR: 1.195, 95% CI: 0.514–2.778, *P* = 0.678). Compared with MHNO without any metabolic abnormality, even obese subjects with only one metabolic abnormality showed a significantly increased the risk of CACS progression (age- and sex-adjusted HR: 2.247, 95% CI: 1.342–3.763, *P* = 0.002). Additional adjustment for HOMA-IR did not attenuated their risk of CACS progression (HR: 2.514, 95% CI: 1.052–6.006, *P* = 0.038). Redefinition of reference group as subjects with normal weight and metabolic health also showed that MHO class II did not increased the risk of CACS progression compared to reference group (S4 Table).

**Table 3:** Risk of CACS progression during follow-up period according to baseline metabolic abnormalities and obesity status.

	N	CACS progression, %	Hazard ratio (HR)			
			HR^a^	*P*-value^a^	HR^b^	*P*-value^b^
MHO definition I						
MHNO (≤1 abnormality)	492	15.9	(reference)	–	(reference)	–
Obesity with ≤1 abnormality (MHO class I)	190	24.2	1.809 (1.255–2.609)	0.001	1.653 (1.144–2.390)	0.007
MHO definition II						
MHNO (no abnormality)	218	11.5	(reference)	–	(reference)	–
Obesity with no abnormality (MHO class II)	58	12.1	1.334 (0.576–3.090)	0.501	1.195 (0.514–2.778)	0.678
Obesity with one abnormality	132	29.5	2.694 (1.622–4.475)	<0.001	2.247 (1.342–3.763)	0.002
Obesity with ≤1 abnormality (MHO class I)	190	24.2	2.324 (1.422–3.800)	0.001	1.972 (1.198–3.248)	0.008

^a^ without adjustment

^b^ with adjustment for age and sex

Abbreviations: CACS, coronary artery calcium score; MHO, metabolically healthy obesity; MHNO, metabolically healthy non-obesity

Among subjects with MHO class I, the risk of CACS progression differed depending whether the subjects maintain or deteriorated the metabolic health during follow-up. The HR of CACS progression was 1.448 (95% CI: 0.921–2.278, *P* = 0.109) in those who maintain metabolic health, but was 2.175 (95% CI: 1.338–3.537, *P* = 0.002) in those who develop metabolic syndrome.

## Discussion

In the current study, we aimed to investigate the influence of MHO on the development of subclinical coronary artery disease using CACS, and the risk of CACS progression by MHO differed according to the definition. MHO class II without any metabolic abnormality did not significantly increase the risk of CACS progression even after adjusting for possible confounders. By contrast, MHO class I, which has been frequently defined in previous studies [24–27,29,30], showed a 1.7-fold higher risk for CACS progression than non-obese metabolically healthy subjects. A stratified analysis of this MHO class I group according to the number of metabolic abnormality (that is, 0 or 1) showed that there was a significant difference in the risk of CACS progression between MHO subjects with no metabolic abnormality and one metabolic abnormality at the baseline. The number of metabolic abnormality at the baseline in MHO class I subjects also determined the risk of developing metabolic syndrome during the follow-up period. Considering the difference in the following risk of CACS progression and developing metabolic syndrome in MHO subjects according to the presence of any metabolic abnormality at the baseline, strict definition of MHO might be needed to differentiate the benign form of obesity from general obese population.

Difference in the definition of MHO might influence the conflicting results on the association between cardiovascular events and MHO. A recent meta-analysis showed that MHO is not a fully benign condition [34]; however, this finding was not consistent [35,36]. There is also conflicting evidence regarding the association between MHO phenotype and subclinical CAD [37–39]. Recently, Chang et al. used a strict definition for MHO, that is, obesity with no metabolic abnormality and low insulin resistance, in a large sample of healthy subjects, and showed that MHO exhibits higher coronary calcification prevalence [37], which is in contrast to ours. Our study showed that, with strict definition, MHO does not increase the risk of subclinical CAD. The difference between two studies might be due to the difference in the baseline cardiovascular risk in both study subjects. Chang et al. included participants with coronary artery calcium deposition (6.8% of study subjects had CACS>0); by contrast, we include only the subjects with no coronary artery calcium deposition (baseline CACS = 0), which suggests low cardiovascular risk [40]. In our preliminary analysis, baseline CACS was not only one of the risk factors for CACS progression, but also correlated to the CT follow-up interval. Therefore, we excluded subjects with baseline CACS > 0 to avoid the confounding effect of baseline CACS and to focus on the development of subclinical CAD. In addition, Chang et al. reported the cross-sectional association between MHO and CACS, which might be considered carefully to compare their result with ours. We assessed the effect of MHO on CAD risk longitudinally through the assessment of change of serial CACS. A dichotomous classification of body weight in our study may have underestimated the risk of MHO; however, a similar result was obtained when the non-obese subject was subdivided into normal weight and overweight.

In our study, during the 45 months of follow-up, 32.2% of MHO class I subjects and 10.2% of MHO class II subjects developed metabolic syndrome. MHO is considered an unstable condition that may deteriorate and transit to MUHO over time. In previous studies that investigated the natural course of MHO, approximately 30–40% of subjects with MHO at baseline developed a metabolically unhealthy state at follow-up [10,29], which was similar to that of MHO class I group in our study. By contrast, using more strict definition of MHO, that is, MHO class II, showed that MHO did not increase the prevalence of metabolic syndrome at follow-up compared to those in non-obese metabolically healthy subjects. Furthermore, MHO class II did not increase the risk of CACS progression, either. Even in the subjects with MHO class I, those maintaining metabolic health during follow-up period did not show an increased risk of CACS progression compared to MHNO. Previous study also showed that among MHO subjects, those who maintained long-term metabolic health did not have higher risk of diabetes and cardiovascular diseases compared to normal weight with no metabolic abnormality [31]. Studies that demonstrated an increased cardiometabolic risk in subjects with the MHO phenotype, including our study, may be partially affected by this metabolic instability of MHO.

There has been no consensus on the definition of MHO, and various definitions have been proposed thus far [2,12,24–27,41]. It is unclear whether the metabolic instability of MHO stems from the intrinsic characteristics of MHO or the absence of a perfect definition of MHO. In the current study, we defined MHO class I following the most frequently used definition in the recent studies [24–27], and confirmed the metabolic instability of MHO class I. The mere absence of metabolic syndrome may not guarantee metabolic health, which contributes to the unstable nature of MHO and higher risk of CAD progression seen in this study. Among subjects with MHO class I, those who showed deteriorated metabolic health at follow-up had significantly higher systolic and diastolic blood pressures and triglyceride level, which are the factors associated with insulin resistance. Ectopic fat [25,42,43] has been suggested as the factor determining the fate of MHO. In our study, there was no difference in the prevalence of abdominal obesity at the baseline according to development of metabolic syndrome at the follow-up. However, among MHO class I subjects, those who developing metabolic syndrome showed a significantly higher level of GGT level compared to those maintaining metabolic health. Although we had no liver fat data of study subjects, GGT level can reflect the liver fat [44]. Liver fat might be more important to the risk of metabolic health than abdominal obesity [42]. High inflammation [24] as well as large ectopic fat [25] also has been reported to be associated with a poor future outcome among MHO subject; however there was no difference in CRP level according to metabolic instability among MHO subjects. The lack of unique criteria for definition is the main barrier to understanding the MHO phenotype, its clinical implications, and the benefits of therapeutic intervention.

The most important strength of our study is that the effect of MHO on CAD risk was assessed longitudinally through the assessment of change of serial CACS. Furthermore, we performed the analysis with homogenous subjects who had a low risk of CAD, that is, CACS = 0 at the baseline, which might reinforce the causal-relationship between MHO and CACS progression shown by our study. Furthermore, the homogenous study population can reduce the confounding effect of the baseline CAD risk in the association between CACS progression and baseline obesity phenotype. It is well-known that a high baseline CAD risk increases future CAD events [40]. The second strength of our study is that we compared the MHO phenotype using comprehensive evaluation during structured health check-ups including medical histories and anthropometric and biochemical measurements. We were able to assess the change in metabolic status during a follow-up period and investigate the influence of metabolic change on the progression of CAD in MHO. Lastly, we applied a stricter definition of MHO, i.e., obesity without any metabolic abnormality (MHO class II). We compared the risk of CACS progression in subjects with MHO class I and class II, and found that the future CAD risk of MHO could be categorized according to the number of metabolic abnormalities combined with obesity.

Despite our important findings, there are some limitations in this study. First, because the study was designed as a retrospective study, confounding factors and bias could be more common than seen in a prospective study. Second, the subjects in our study underwent CACS on request, which might be a source of selection bias. Time interval between baseline and follow-up CACS measurement might be also partly dependent on individuals’ demand. Lastly, this study was performed in Korea, and therefore, its results cannot be generalized to other ethnicities.

In conclusion, the definition of MHO is of importance when evaluating the influence of MHO on the risk of CAD progression. With the more commonly used definition (MHO class I), MHO seemed to be harmful and exhibited a significant risk of subclinical CAD development. The risks of metabolic deterioration and CACS progression were significant in MHO class I subjects, and metabolic instability seems to partly contribute to CACS progression in these population. On the other hand, MHO class II was not associated with the risk of developing metabolic syndrome and CACS progression. A stricter definition of MHO might help differentiate the benign phenotype of obesity from the other phenotypes; however, longer term effect should be explored in the future study.

## Supporting information

S1 Fig The prevalence of metabolic syndrome during the follow-up. The prevalence of metabolic syndrome at the follow-up was CACS progression during the follow-up period was 10.8%, 21.6%, 48.9%, 62.5%, and 83.3% in non-obese subjects with 0, 1, 2, 3, and 4 metabolic abnormalities, respectively (black bars; *P* for trend < 0.001), and 10.2%, 41.0%, 61.3%, 67.1%, and 86.4%in obese subjects with 0, 1, 2, 3, and 4 metabolic abnormalities, respectively (gray bars; *P* for trend < 0.001).
(TIF)

S2 Fig The prevalence of coronary artery calcium score (CACS) progression during the follow-up period. The prevalence of CACS progression during the follow-up period was 11.5%, 19.3%, 28.6%, 32.7%, and 33.3% in non-obese subjects with 0, 1, 2, 3, and 4 metabolic abnormalities, respectively (black bars; *P* for trend < 0.001), and 12.1%, 29.5%, 25.3%, 37.9%, and 28.0% in obese subjects with 0, 1, 2, 3, and 4 metabolic abnormalities, respectively (gray bars; *P* for trend = 0.018).
(TIF)

S1 Table Clinical characteristics according to baseline metabolic abnormality and obesity. (DOCX)

S2 Table Risk of developing metabolic syndrome during follow-up according to BMI categories. (DOCX)

S3 Table Comparison of the characteristics between metabolically stable and deteriorated subjects in MHO class I. (DOCX)

S4 Table Risk of CACS progression during follow-up period according to BMI categories. (DOCX)

## References

[1] Bastien M, Poirier P, Lemieux I, Despres JP. Overview of epidemiology and contribution of obesity to cardiovascular disease. Prog Cardiovasc Dis 2014; 56(4): 369–381. 10.1016/j.pcad.2013.10.016 24438728

[2] Wildman RP, Muntner P, Reynolds K, McGinn AP, Rajpathak S, Wylie-Rosett J, et al The obese without cardiometabolic risk factor clustering and the normal weight with cardiometabolic risk factor clustering: prevalence and correlates of 2 phenotypes among the US population (NHANES 1999–2004). Arch Intern Med 2008; 168(15): 1617–1624. 10.1001/archinte.168.15.1617 18695075

[3] Stefan N, Haring HU, Hu FB, Schulze MB. Metabolically healthy obesity: epidemiology, mechanisms, and clinical implications. Lancet Diabetes Endocrinol 2013; 1(2): 152–162. 10.1016/S2213-8587(13)70062-7 24622321

[4] Stefan N, Kantartzis K, Machann J, Schick F, Thamer C, Rittig K, et al Identification and characterization of metabolically benign obesity in humans. Arch Intern Med 2008; 168(15): 1609–1616. 10.1001/archinte.168.15.1609 18695074

[5] Karelis AD, Rabasa-Lhoret R. Obesity: Can inflammatory status define metabolic health? Nat Rev Endocrinol 2013; 9(12): 694–695. 10.1038/nrendo.2013.198 24126480

[6] Lionetti L, Mollica MP, Lombardi A, Cavaliere G, Gifuni G, Barletta A. From chronic overnutrition to insulin resistance: the role of fat-storing capacity and inflammation. Nutr Metab Cardiovasc Dis 2009; 19(2): 146–152. 10.1016/j.numecd.2008.10.010 19171470

[7] Kim JY, van de Wall E, Laplante M, Azzara A, Trujillo ME, Hofmann SM, et al Obesity-associated improvements in metabolic profile through expansion of adipose tissue. J Clin Invest 2007; 117(9): 2621–2637. 10.1172/JCI31021 17717599 PMC1950456

[8] Kusminski CM, Holland WL, Sun K, Park J, Spurgin SB, Lin Y, et al MitoNEET-driven alterations in adipocyte mitochondrial activity reveal a crucial adaptive process that preserves insulin sensitivity in obesity. Nat Med 2012; 18(10): 1539–1549. 10.1038/nm.2899 22961109 PMC3745511

[9] Herman MA, Peroni OD, Villoria J, Schon MR, Abumrad NA, Bluher M, et al A novel ChREBP isoform in adipose tissue regulates systemic glucose metabolism. Nature 2012; 484(7394): 333–338. 10.1038/nature10986 22466288 PMC3341994

[10] Eshtiaghi R, Keihani S, Hosseinpanah F, Barzin M, Azizi F. Natural course of metabolically healthy abdominal obese adults after 10 years of follow-up: the Tehran Lipid and Glucose Study. Int J Obes (Lond) 2014. 10.1038/ijo.2014.176 25287753

[11] Arnlov J, Sundstrom J, Ingelsson E, Lind L. Impact of BMI and the metabolic syndrome on the risk of diabetes in middle-aged men. Diabetes Care 2011; 34(1): 61–65. 10.2337/dc10-0955 20852030 PMC3005442

[12] Meigs JB, Wilson PW, Fox CS, Vasan RS, Nathan DM, Sullivan LM, et al Body mass index, metabolic syndrome, and risk of type 2 diabetes or cardiovascular disease. J Clin Endocrinol Metab 2006; 91(8): 2906–2912. 10.1210/jc.2006-0594 16735483

[13] Hamer M, Stamatakis E. Metabolically healthy obesity and risk of all-cause and cardiovascular disease mortality. J Clin Endocrinol Metab 2012; 97(7): 2482–2488. 10.1210/jc.2011-3475 22508708 PMC3387408

[14] Song Y, Manson JE, Meigs JB, Ridker PM, Buring JE, Liu S. Comparison of usefulness of body mass index versus metabolic risk factors in predicting 10-year risk of cardiovascular events in women. Am J Cardiol 2007; 100(11): 1654–1658. 10.1016/j.amjcard.2007.06.073 18036364 PMC2098701

[15] El Khoudary SR, Shin C, Masaki K, Miura K, Budoff M, Edmundowicz D, et al Ectopic cardiovascular fat in middle-aged men: effects of race/ethnicity, overall and central adiposity. The ERA JUMP study. Int J Obes (Lond) 2014. 10.1038/ijo.2014.154 PMC4324390 25109783

[16] Simons DB, Schwartz RS, Edwards WD, Sheedy PF, Breen JF, Rumberger JA. Noninvasive definition of anatomic coronary artery disease by ultrafast computed tomographic scanning: a quantitative pathologic comparison study. J Am Coll Cardiol 1992; 20(5): 1118–1126. 1401612 10.1016/0735-1097(92)90367-v

[17] Bonow RO. Clinical practice. Should coronary calcium screening be used in cardiovascular prevention strategies? N Engl J Med 2009; 361(10): 990–997. 10.1056/NEJMcp0902177 19726773

[18] Raggi P, Cooil B, Shaw LJ, Aboulhson J, Takasu J, Budoff M, et al Progression of coronary calcium on serial electron beam tomographic scanning is greater in patients with future myocardial infarction. Am J Cardiol 2003; 92(7): 827–829. 14516885 10.1016/s0002-9149(03)00892-0

[19] Raggi P, Callister TQ, Shaw LJ. Progression of coronary artery calcium and risk of first myocardial infarction in patients receiving cholesterol-lowering therapy. Arterioscler Thromb Vasc Biol 2004; 24(7): 1272–1277. 10.1161/01.ATV.0000127024.40516.ef 15059806

[20] McEvoy JW, Blaha MJ, Defilippis AP, Budoff MJ, Nasir K, Blumenthal RS, et al Coronary artery calcium progression: an important clinical measurement? A review of published reports. J Am Coll Cardiol 2010; 56(20): 1613–1622. 10.1016/j.jacc.2010.06.038 21050970

[21] Budoff MJ, Hokanson JE, Nasir K, Shaw LJ, Kinney GL, Chow D, et al Progression of coronary artery calcium predicts all-cause mortality. JACC Cardiovasc Imaging 2010; 3(12): 1229–1236. 10.1016/j.jcmg.2010.08.018 21163451

[22] Matthews DR, Hosker JP, Rudenski AS, Naylor BA, Treacher DF, Turner RC. Homeostasis model assessment: insulin resistance and beta-cell function from fasting plasma glucose and insulin concentrations in man. Diabetologia 1985; 28(7): 412–419. 3899825 10.1007/BF00280883

[23] World Health Organization. The Asia-Pacific perspective: redefining obesity and its treatment. 2000.

[24] Jung CH, Lee MJ, Kang YM, Jang JE, Leem J, Hwang JY, et al The risk of incident type 2 diabetes in a Korean metabolically healthy obese population: the role of systemic inflammation. J Clin Endocrinol Metab 2015; 100(3): 934–941. 10.1210/jc.2014-3885 25490279

[25] Heianza Y, Arase Y, Tsuji H, Fujihara K, Saito K, Hsieh SD, et al Metabolically healthy obesity, presence or absence of fatty liver, and risk of type 2 diabetes in Japanese individuals: Toranomon Hospital Health Management Center Study 20 (TOPICS 20). J Clin Endocrinol Metab 2014; 99(8): 2952–2960. 10.1210/jc.2013-4427 24823457

[26] Hinnouho GM, Czernichow S, Dugravot A, Nabi H, Brunner EJ, Kivimaki M, et al Metabolically healthy obesity and the risk of cardiovascular disease and type 2 diabetes: the Whitehall II cohort study. Eur Heart J 2015; 36(9): 551–559. 10.1093/eurheartj/ehu123 24670711 PMC4344958

[27] Eshtiaghi R, Keihani S, Hosseinpanah F, Barzin M, Azizi F. Natural course of metabolically healthy abdominal obese adults after 10 years of follow-up: the Tehran Lipid and Glucose Study. Int J Obes (Lond) 2015; 39(3): 514–519. 25287753 10.1038/ijo.2014.176

[28] Third Report of the National Cholesterol Education Program (NCEP) Expert Panel on Detection, Evaluation, and Treatment of High Blood Cholesterol in Adults (Adult Treatment Panel III) final report. Circulation 2002; 106(25): 3143–3421. 12485966

[29] Appleton SL, Seaborn CJ, Visvanathan R, Hill CL, Gill TK, Taylor AW, et al Diabetes and cardiovascular disease outcomes in the metabolically healthy obese phenotype: a cohort study. Diabetes Care 2013; 36(8): 2388–2394. 10.2337/dc12-1971 23491523 PMC3714523

[30] Heianza Y, Kato K, Kodama S, Suzuki A, Tanaka S, Hanyu O, et al Stability and changes in metabolically healthy overweight or obesity and risk of future diabetes: Niigata wellness study. Obesity (Silver Spring) 2014; 22(11): 2420–2425. 25131796 10.1002/oby.20855

[31] van Vliet-Ostaptchouk JV, Nuotio ML, Slagter SN, Doiron D, Fischer K, Foco L, et al The prevalence of metabolic syndrome and metabolically healthy obesity in Europe: a collaborative analysis of ten large cohort studies. BMC Endocr Disord 2014; 149. 10.1186/1472-6823-14-9 PMC3923238 24484869

[32] Agatston AS, Janowitz WR, Hildner FJ, Zusmer NR, Viamonte M Jr., Detrano R. Quantification of coronary artery calcium using ultrafast computed tomography. J Am Coll Cardiol 1990; 15(4): 827–832. 2407762 10.1016/0735-1097(90)90282-t

[33] Berry JD, Liu K, Folsom AR, Lewis CE, Carr JJ, Polak JF, et al Prevalence and progression of subclinical atherosclerosis in younger adults with low short-term but high lifetime estimated risk for cardiovascular disease: the coronary artery risk development in young adults study and multi-ethnic study of atherosclerosis. Circulation 2009; 119(3): 382–389. 10.1161/CIRCULATIONAHA.108.800235 19139385 PMC2836265

[34] Aung K, Lorenzo C, Hinojosa MA, Haffner SM. Risk of developing diabetes and cardiovascular disease in metabolically unhealthy normal-weight and metabolically healthy obese individuals. J Clin Endocrinol Metab 2014; 99(2): 462–468. 10.1210/jc.2013-2832 24257907 PMC3913817

[35] Kramer CK, Zinman B, Retnakaran R. Are metabolically healthy overweight and obesity benign conditions?: A systematic review and meta-analysis. Ann Intern Med 2013; 159(11): 758–769. 10.7326/0003-4819-159-11-201312030-00008 24297192

[36] Harrington M, Gibson S, Cottrell RC. A review and meta-analysis of the effect of weight loss on all-cause mortality risk. Nutr Res Rev 2009; 22(1): 93–108. 10.1017/S0954422409990035 19555520

[37] Chang Y, Kim BK, Yun KE, Cho J, Zhang Y, Rampal S, et al Metabolically-healthy obesity and coronary artery calcification. J Am Coll Cardiol 2014; 63(24): 2679–2686. 10.1016/j.jacc.2014.03.042 24794119

[38] Rhee EJ, Seo MH, Kim JD, Jeon WS, Park SE, Park CY, et al Metabolic health is more closely associated with coronary artery calcification than obesity. PLoS One 2013; 8(9): e74564 10.1371/journal.pone.0074564 24040286 PMC3770589

[39] Jung CH, Lee MJ, Hwang JY, Jang JE, Leem J, Yang DH, et al Association of metabolically healthy obesity with subclinical coronary atherosclerosis in a Korean population. Obesity (Silver Spring) 2014; 22(12): 2613–2620. 25155902 10.1002/oby.20883

[40] Budoff MJ, Achenbach S, Blumenthal RS, Carr JJ, Goldin JG, Greenland P, et al Assessment of coronary artery disease by cardiac computed tomography: a scientific statement from the American Heart Association Committee on Cardiovascular Imaging and Intervention, Council on Cardiovascular Radiology and Intervention, and Committee on Cardiac Imaging, Council on Clinical Cardiology. Circulation 2006; 114(16): 1761–1791. 10.1161/CIRCULATIONAHA.106.178458 17015792

[41] Lynch LA, O'Connell JM, Kwasnik AK, Cawood TJ, O'Farrelly C, O'Shea DB. Are natural killer cells protecting the metabolically healthy obese patient? Obesity (Silver Spring) 2009; 17(3): 601–605. 19238145 10.1038/oby.2008.565

[42] Stefan N. Nonalcoholic Fatty Liver Disease and Coronary Artery Calcification. Clin Gastroenterol Hepatol 2016; 14(9): 1345–1346. 10.1016/j.cgh.2016.05.025 27237428

[43] Stefan N, Fritsche A, Schick F, Haring HU. Phenotypes of prediabetes and stratification of cardiometabolic risk. Lancet Diabetes Endocrinol 2016; 4(9): 789–798. 10.1016/S2213-8587(16)00082-6 27185609

[44] Volzke H, Alte D, Ittermann T, Schmidt CO, Rettig R, Mayerle J, et al Subjects with sonographical hepatic steatosis should be excluded from studies to establish upper reference levels of serum transaminases. Liver Int 2011; 31(7): 985–993. 10.1111/j.1478-3231.2010.02371.x 21078068

