# Supplementary figures and images for: Influence of the definition of “metabolically healthy obesity” on the progression of coronary artery calcification
Source: PLoS One. 2017 Jun 2;12(6):e0178741. doi: 10.1371/journal.pone.0178741 (PMC5456095; doi:10.1371/journal.pone.0178741)

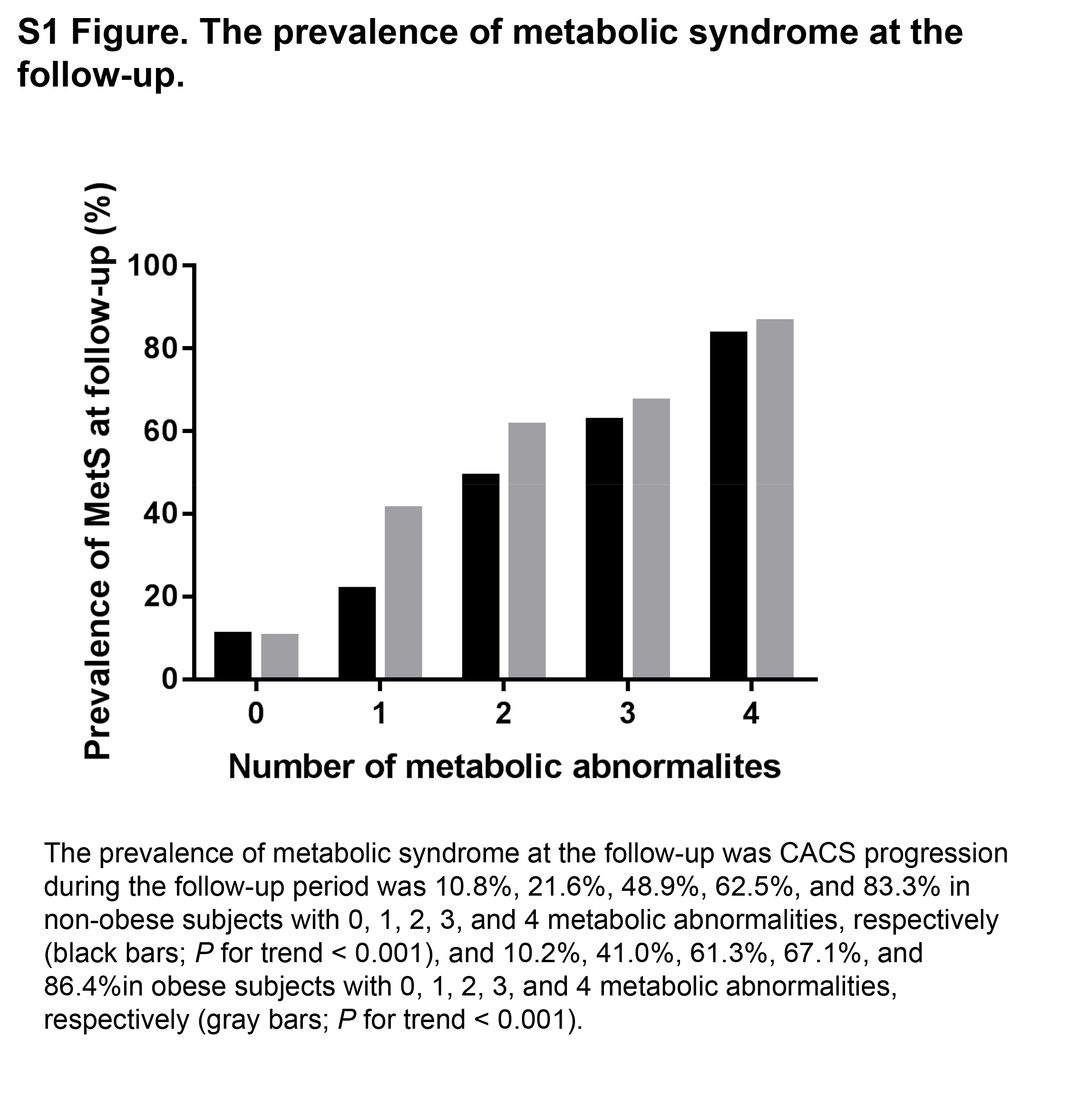

Supplement: S1 Fig — The prevalence of metabolic syndrome at the follow-up was CACS progression during the follow-up period was 10.8%, 21.6%, 48.9%, 62.5%, and 83.3% in non-obese subjects with 0, 1, 2, 3, and 4 metabolic abnormalities, respectively (black bars; P for trend < 0.001), and 10.2%, 41.0%, 61.3%, 67.1%, and 86.4%in obese subjects with 0, 1, 2, 3, and 4 metabolic abnormalities, respectively (gray bars; P for trend < 0.001). (TIF) [file pone.0178741.s001.tif]

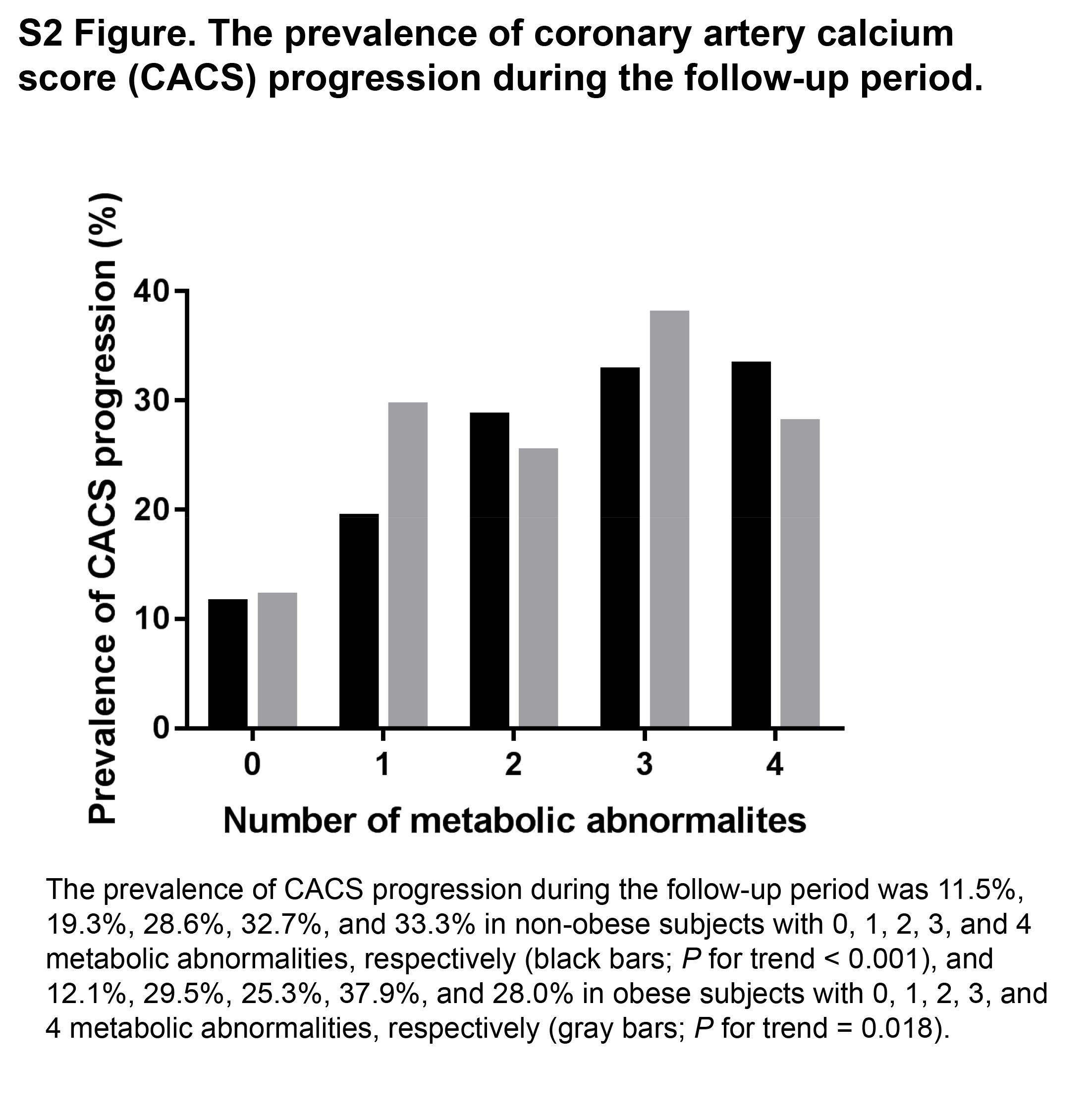

Supplement: S2 Fig — The prevalence of CACS progression during the follow-up period was 11.5%, 19.3%, 28.6%, 32.7%, and 33.3% in non-obese subjects with 0, 1, 2, 3, and 4 metabolic abnormalities, respectively (black bars; P for trend < 0.001), and 12.1%, 29.5%, 25.3%, 37.9%, and 28.0% in obese subjects with 0, 1, 2, 3, and 4 metabolic abnormalities, respectively (gray bars; P for trend = 0.018). (TIF) [file pone.0178741.s002.tif]
