# Supplementary material for: Influence of the definition of “metabolically healthy obesity” on the progression of coronary artery calcification
Source: PLoS One. 2017 Jun 2;12(6):e0178741. doi: 10.1371/journal.pone.0178741 (PMC5456095; doi:10.1371/journal.pone.0178741)
Supplement: S1 Table — (DOCX) [file pone.0178741.s003.docx]

**S1 Table.** Clinical characteristics according to baseline metabolic abnormality and obesity

|  | | MHNO | | MHO | |  |  |  |
| --- | --- | --- | --- | --- | --- | --- | --- | --- |
| Number of  metabolic abnormality | | 0 | 0 | | 1 | *P*-value^a^ | *P*-value^b^ | *P*-value^c^ |
| N | | 218 | 58 | | 132 |  |  |  |
| Male, % | | 59.2 | 75.9 | | 85.6 | 0.020 | <0.001 | 0.428 |
| Age, years | | 54.3± 6.6 | 54.6 ± 6.8 | | 54.6 ± 6.9 | 0.758 | 0.697 | 0.992 |
| BMI^*^, kg/m^2^ | | 22.3±1.8 | 26.4± 1.2 | | 26.5± 1.4 | <0.001 | <0.001 | 0.368 |
| WC^*^, cm | M | 84.3±5.1 | 92.4±4.7 | | 92.3 ±4.3 | <0.001 | <0.001 | 0.842 |
|  | F | 79.1±5.8 | 89.1±4.1 | | 92.3±6.4 | <0.001 | <0.001 | 0.148 |
| Systolic BP, mmHg | | 109.1±10.2 | 112.6± 8.7 | | 122± 14 | 0.019 | <0.001 | <0.001 |
| Diastolic BP, mmHg | | 70.5± 8.3 | 72.9± 6.8 | | 80 ± 10 | 0.020 | <0.001 | <0.001 |
| FPG, mmol/L | | 5.0±0.3 | 5.1± 0.3 | | 5.7± 0.4 | 0.011 | <0.001 | <0.001 |
| HbA1c, % | | 5.6± 0.3 | 5.6± 0.3 | | 5.7± 0.4 | 0.366 | 0.026 | 0.334 |
| HDL-C, mmol/L | M | 1.39± 0.26 | 1.39± 0.27 | | 1.33± 0.29 | 0.804 | 0.101 | 0.359 |
|  | F | 1.66±0.33 | 1.48± 0.17 | | 1.41± 0.32 | 0.003 | 0.002 | 0.456 |
| Triglyceride, mmol/L^*^ | | 0.92±0.33 | 1.04±0.33 | | 1.29±0.66 | 0.012 | <0.001 | 0.010 |
| HOMA-IR^*^ | | 1.70±1.76 | 2.06± 1.39 | | 2.60± 1.36 | 0.162 | <0.001 | 0.015 |
| hs-CRP^*^ , mg/L | | 1.1 ± 2.5 | 1.4 ±2.4 | | 2.0± 4.6 | 0.249 | 0.001 | 0.197 |
| Diabetes mellitus, % | | 0.0 | 0.0 | | 4.5 | — | <0.001 | 0.099 |
| Hypertension, % | | 0.0 | 0.0 | | 32.6 | — | <0.001 | <0.001 |
| Abdominal obesity, % | | 27.1 | 78.2 | | 76.2 | <0.001 | <0.001 | 0.765 |
| Current smoker, % | | 15.2 | 28.2 | | 13.3 | 0.060 | 0.686 | 0.043 |
| Exercise, % | | 25.7 | 22.4 | | 33.3 | 0.609 | 0.125 | 0.130 |

*Log-transformed when comparing among groups

^a^Compared between MHNO and MHO with no metabolic abnormality

^b^Compared between MHNO and MHO with one metabolic abnormality

^c^Compared between MHO with and without a metabolic abnormality

Abbreviations: MHNO, metabolically healthy and non-obesity; MHO, metabolically healthy obesity
