# Supplementary material for: Influence of the definition of “metabolically healthy obesity” on the progression of coronary artery calcification
Source: PLoS One. 2017 Jun 2;12(6):e0178741. doi: 10.1371/journal.pone.0178741 (PMC5456095; doi:10.1371/journal.pone.0178741)
Supplement: S2 Table — (DOCX) [file pone.0178741.s004.docx]

**S2 Table.** Risk of developing metabolic syndrome during follow-up according to BMI categories

|  | Metabolic syndrome^a^ at follow-up | | | |
| --- | --- | --- | --- | --- |
|  | Hazzard ratio^e^ | *P*-value^e^ | Hazzard ratio^f^ | *P*-value^f^ |
| MHO definition I |  |  |  |  |
| Normal weight^b^ | (reference) |  | (reference) |  |
| Overweight^c^ | 1.373 (0.849–2.222) | 0.197 | 1.250 (0.767–2.036) | 0.371 |
| Obesity^d^ | 2.750 (1.769–4.275) | <0.001 | 2.437 (1.551–3.830) | <0.001 |
| MHO definition II |  |  |  |  |
| Normal weight^b^ | (reference) |  | (reference) |  |
| Overweight^c^ | 1.807 (0.744–4.392) | 0.192 | 1.883 (0.727–4.878) | 0.193 |
| Obesity^d^ | 1.521 (0.507–4.568) | 0.455 | 1.509 (0.487–4.681) | 0.476 |

Underweight was excluded for the current analysis as statistical power was inappropriate due to the small number of subjects with underweight.

^a^those who met ≥ 2 of the following National Cholesterol Education Program–Adult Treatment Panel III criteria except abdominal obesity criterion

^b^BMI≥ 18.5 kg/m^2^ and <23 kg/m^2^

^c^BMI ≥ 23 kg/m^2^ and <25 kg/m^2^

^d^BMI ≥25 kg/m^2^

^e^without adjustment

^f^ with adjustment for age and sex

Abbreviations: MHO, metabolically healthy obesity; BMI, body mass index
