# Supplementary material for: Influence of the definition of “metabolically healthy obesity” on the progression of coronary artery calcification
Source: PLoS One. 2017 Jun 2;12(6):e0178741. doi: 10.1371/journal.pone.0178741 (PMC5456095; doi:10.1371/journal.pone.0178741)
Supplement: S3 Table — (DOCX) [file pone.0178741.s005.docx]

**S3 Table.** Comparison of the characteristics between metabolically stable and deteriorated subjects in MHO class I

|  | No MetS^a^ at follow-up | MetS^a^ at follow-up | *P*-value |
| --- | --- | --- | --- |
| Age (yr) | 54.5 ± 6.4 | 53.7 ± 6.8 | 0.811 |
| Sex (male, %) | 90.5 | 87.3 | 0.595 |
| Body mass index^*^ (kg/m^2^) | 26.5 ± 1.3 | 26.6 ± 1.4 | 0.597 |
| SBP (mmHg) | 117.3 ± 12.0 | 123.5 ± 13.0 | 0.002 |
| DBP (mmHg) | 76.8 ± 8.8 | 81.9 ± 9.4 | <0.001 |
| Fasting blood glucose (mmol/L) | 5.3 ± 0.6 | 5.4± 0.6 | 0.425 |
| HbA1c (%) | 5.7 ± 0.3 | 5.6 ±0.4 | 0.571 |
| LDL-cholesterol (mmol/L) | 3.1 ±0.8 | 3.4 ± 0.8 | 0.083 |
| Triglyceride^*^ (mmol/L) | 1.2 ± 0.5 | 1.4 ± 0.8 | 0.040 |
| AST^*^, IU/L | 26.4 ± 10.3 | 25.6 ± 7.3 | 0.834 |
| ALT^*^, IU/L | 29.6 ± 16.0 | 33.6 ± 18.3 | 0.099 |
| GGT^*^, IU/L | 37.4 ± 24.3 | 51.4 ± 41.8 | 0.006 |
| HOMA-IR^*^ | 2.24 ±1.09 | 2.69 ±1.54 | 0.155 |
| CRP^*^, mg/L | 1.3 ± 2.3 | 1.7 ± 2.8 | 0.614 |
| Abdominal obesity (%) | 73.5 | 78.2 | 0.572 |
| Hypertension (%) | 15.5 | 36.4 | 0.003 |
| Diabetes mellitus (%) | 2.6 | 5.5 | 0.388 |
| Smoking (%) | 20.0 | 18.9 | 0.999 |

Data was presented as mean ± standard deviation

^*^Log-transformed when comparing among groups

^a^Those who met ≥ 2 of the following National Cholesterol Education Program–Adult Treatment Panel III criteria except abdominal obesity criterion

Abbreviations: MHO, metabolically healthy obesity; MetS, metabolic syndrome; SBP, systolic blood pressure; DBP, diastolic blood pressure; LDL-C, low density lipoprotein cholesterol; AST, aspartate aminotransferase; ALT, alanine aminotransferase; GGT, gamma-glutamyl transferase; HOMA-IR, homeostatic model assessment of insulin resistance; CRP, c-reactive protein.
