# Supplementary material for: Influence of the definition of “metabolically healthy obesity” on the progression of coronary artery calcification
Source: PLoS One. 2017 Jun 2;12(6):e0178741. doi: 10.1371/journal.pone.0178741 (PMC5456095; doi:10.1371/journal.pone.0178741)
Supplement: S4 Table — (DOCX) [file pone.0178741.s006.docx]

**S4 Table.** Risk of CACS progression during follow-up period according to BMI categories

|  | Hazard ratio (HR) | | | |
| --- | --- | --- | --- | --- |
|  | HR^d^ | *P*-value^d^ | HR^e^ | *P*-value^e^ |
| MHO definition I |  |  |  |  |
| Normal weight^a^ | (reference) |  | (reference) |  |
| Overweight^b^ | 1.260 (0.804 – 1.973) | 0.314 | 0.996 (0.629 – 1.577) | 0.985 |
| Obesity^c^ | 2.017 (1.315 – 3.095) | 0.001 | 1.652 (1.067 – 2.558) | 0.025 |
| MHO definition II |  |  |  |  |
| Normal weight^a^ | (reference) |  | (reference) |  |
| Overweight^b^ | 1.256 (0.561 – 2.813) | 0.579 | 0.743 (0.294 – 1.877) | 0.530 |
| Obesity^c^ | 1.532 (0.618 – 3.793) | 0.357 | 1.149 (0.441 – 2.996) | 0.776 |

Underweight was excluded for the current analysis as statistical power was inappropriate due to the small number of subjects with underweight.

^a^BMI≥ 18.5 kg/m^2^ and <23 kg/m^2^

^b^BMI ≥ 23 kg/m^2^ and <25 kg/m^2^

^c^BMI ≥25 kg/m^2^

^d^without adjustment

^e^ with adjustment for age and sex

Abbreviations: CACS, coronary artery calcium score; BMI, body mass index; MHO, metabolically healthy obesity
